# Determining the content and needs assessment a mobile-based self-care program in infertile men

**DOI:** 10.1186/s12911-023-02366-2

**Published:** 2023-11-13

**Authors:** Azadeh Nadjarzadeh, Alireza Fallahzadeh, Arezoo Abasi, Mohammad Mehdi Poornematy, Hamid Reza Farahzadi, Seyed Ali Fatemi Aghda

**Affiliations:** 1 Nutrition and Food Security Research Center, Shahid Sadoughi University of Medical Sciences, Yazd, Iran; 2 Department of Nutrition, School of Public Health, Shahid Sadoughi University of Medical Sciences, Yazd, Iran; 3 Research Center for Health Technology Assessment and Medical Informatics, School of Public Health, Shahid Sadoughi University of Medical Sciences, Yazd, Iran; 4 Department of Health Information Management, School of Health Management and Information Sciences, Iran University of Medical Sciences, Tehran, Iran

**Keywords:** Infertility, Self-care, Educational needs Assessment, Male infertility, Mhealth

## Abstract

**Background:**

Infertility is a public health problem in the world, using new technology, such as mobile phones, is increasing in the field of health. This study aimed to determine the Necessity of self-care training contents by performing a needs analysis among men with infertility problems to design a mobile phone-based application.

**Methods:**

Followed by reviewing the related literature, a questionnaire including 40 educational items and seven software features was designed in three general sections and distributed among 30 specialists in nutrition (n = 18) and infertility (n = 12). The validity of the questionnaire was confirmed by a panel of experts in nutrition, infertility, and medical informatics. The questionnaire’s reliability was also corroborated by Cronbach’s alpha of 86.4.

**Results:**

All items related to the software features and most items in the questionnaire were deemed necessary by participants. However, the items: “Occupation and history of chronic diseases” in the demographic information section and “Effects of infertility and food allergy” in the educational section were not confirmed.

**Conclusion:**

The present findings could not only highlight the patients’ roles in managing their disease but also increase the healthcare workers’ awareness in designing the hospital information system.

**Supplementary Information:**

The online version contains supplementary material available at 10.1186/s12911-023-02366-2.

## Background

Infertility is a medical condition, referring to the inability to conceive after one year of regular unprotected intercourse without using contraceptive methods, which is recognized as a global public health problem [1]. According to various studies, about 50 to 80 million people suffer from infertility and eight to 12% of couples are affected by infertility and its related complications worldwide [2]. In developing countries, the prevalence of infertility is higher than the global statistics; for instance, more than two million couples suffer from infertility in Iran [3]. Although infertility is reported almost equally between men and women and no significant difference is observed between the two genders in this regard, this problem has often been attributed to women, so that men’s characteristics of fertility behavior have been ignored or considered similar to those of women [4, 5].

However, the causes of male infertility are widely various, including obesity, older age, DNA damage caused by common medical conditions such as cancer, diabetes, obesity, varicocele and genital tract infections, lifestyle factors such as smoking, consumption of alcohol, and abuse of drugs (cocaine and anabolic steroids) [6–9]. Regular physical activity not only aids individuals to maintain and improve their body fitness but also plays a significant role in fertility [10, 11]. Obesity, along with high blood pressure and diabetes, is known as a risk factor for male infertility. Given the global prevalence of obesity, special attention has been paid to the relationship between infertility and obesity [12, 13]. In this regard, varicocele (with a prevalence of 40%) has been introduced as one of the most prevalent but preventable and curable causes of infertility followed by congenital and genetic factors among men [14].

Age (especially in women), black race, low education, single status, and rural residence were determined as effective demographic factors affecting infertility. Among social factors, economic uncertainty, lack of family support policies, job security, as well as access to quality and affordable childcare affect the treatment process [6, 15–17].

Infertility and its treatment stages are deemed stressful challenges with significant negative social impacts on the lives of infertile couples causing them to experience feelings and challenges, such as depression, emotional stress, anxiety, violence, social stigma, low self-esteem, and even divorce. In some cases, the fear of infertility has even caused couples to abandon contraceptive methods in order to prove their fertility from a social point of view [18–20].

Infertility treatment entails a long-term process that may take months or even years without any guarantee of success. In this regard, the lack of access to reliable information and the low quality of the available information are among the greatest challenges in most countries, including Iran in dealing with infertility [18, 21]. Based on the literature [22–24], only half of the couples have enough information about infertility and men are at lower levels of information than women. Due to cultural and social conditions, men usually refuse to receive information from health professionals. To meet this problem, using online resources and e-health can be beneficial for men due to their anonymity and accessibility.

In a randomized controlled trial study, Schick et al. investigated the role of supporting couples undergoing infertility treatment using mobile phones. In this study, the intervention group was sent daily messages with a positive attitude. Considering the stress of these couples and the unavailability of counselors in every place, the use of mobile phone training was useful and the results and consequences were reported to be effective and useful [25].

Malekpour et al. conducted a study with the aim of investigating the effectiveness of a lifestyle for couples undergoing assisted reproductive technology (ART). The results of the study showed that the lifestyle training program including diet, physical activity, sleep disorders, sleep hygiene, meal planning, stress and anxiety management methods is effective among patients [26].

Information technology and applications are desirable tools for improving awareness and educational levels in meeting the extensive needs of health services for specialized groups [21]. Electronic health has provided health information online and led to meeting the information needs of patients in populations that have difficulty accessing treatment [27]. The popularity of smartphones is increasing worldwide and this rate is higher among young and middle-aged people than the elderly [28, 29]. Since infertile couples are often from the young age group, the use of mobile health (mHealth) can be of great importance for this group by reducing health inequality [30]. Such educational interventions have improved the quality of life in patients with cancer and diabetes [31–33].

Given the scarcity of information on the educational content related to infertility among men and the fact that educational courses have mainly been focused on women, men are at lower levels of knowledge of fertility. Moreover, men are more inclined to obtain their fertility information needs from available and anonymous sources to reduce their feelings of worthlessness and inadequacy (a threat to their sense of masculinity). Due to the increase and popularity of mobile phones as well as the unavailability of sufficient and reliable information, the present study was carried out to determine the minimum educational requirement of a mobile phone-based self-care program for men with infertility problems. Since no study has ever tackled this topic in Iran, However, there are studies in other countries [25, 26, 34] that are in line with the purpose of the study and consider the necessity of this work in view of the culture and manners of each nation. the findings can be helpful in maintaining the spirit and sense of masculinity in male patients with infertility.

## Methods

**Fig. 1:**
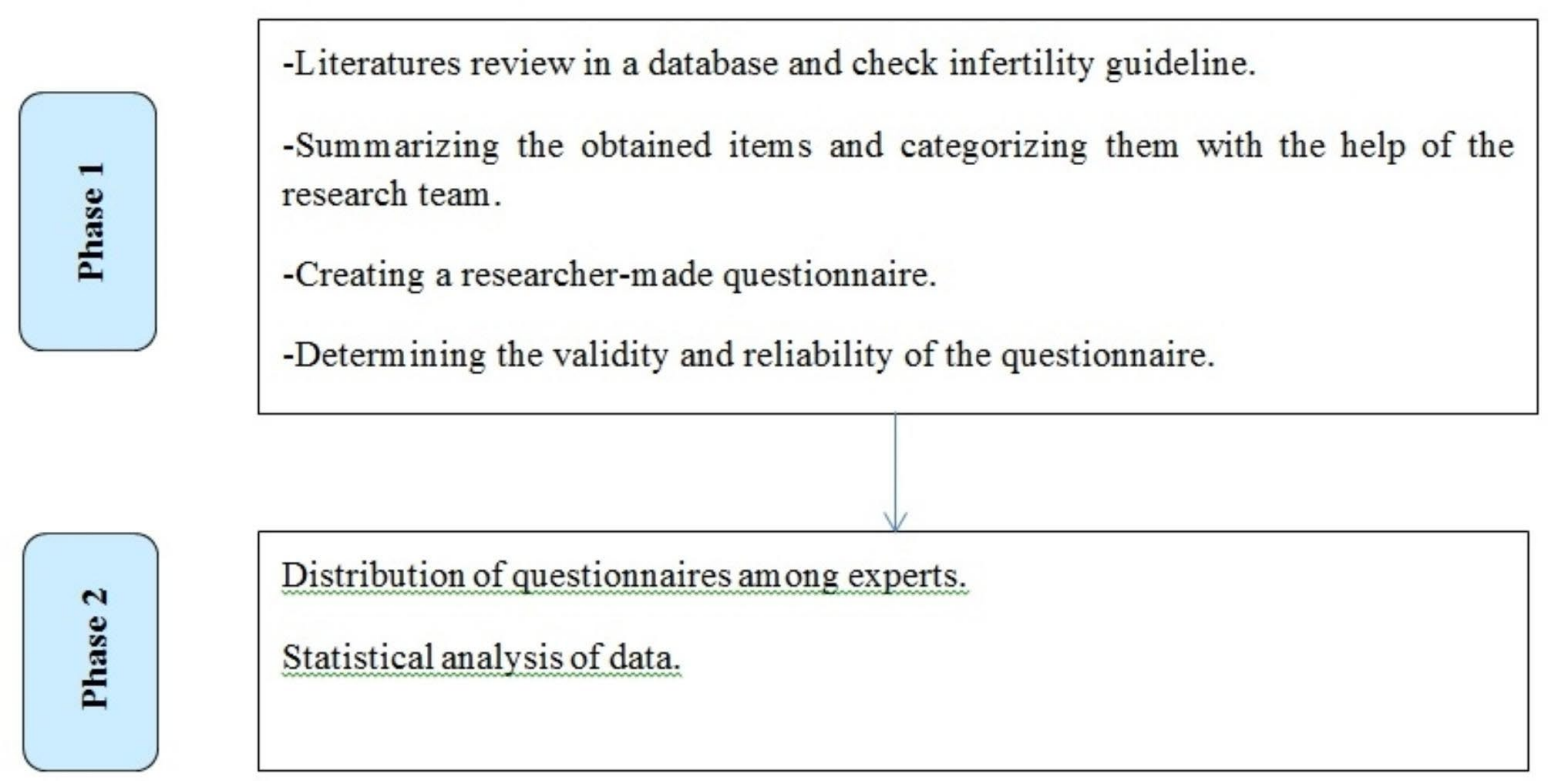
Workflow diagram of study

This study was conducted in two phases (Fig. 1).

In the first phase, the related literature was searched to find articles and publications related to the research questions. As a result, a researcher-made questionnaire was designed.

To determine the important and decisive educational content needed for the application, previous articles and publications were navigated using the keywords “self-care, needs assessment, male infertility, and minimum data” in scientific databases of PubMed, Scopus, Science Direct, and Medline from 2000 to 2020. Considering the research environment and the impact of genetic and geographical location on infertility, reliable Iranian databases (Meg Iran, Jihad Academic) were also investigated. Later, a questionnaire was designed based on the obtained results and the research advisor’s opinions. The respondents were supposed to read each item and rate its necessity on a 5-point Likert scale from very low (1 score), low (2 scores), moderate (3 scores), high (4 scores), to very high (5 scores). This questionnaire includes the participants’ demographic information (5 questions), patient’s demographic information (9 questions), required educational contents to be included in the application (40 questions), and features of the application software (7 questions) and Suggestions section.

The educational content section covered topics on the main disease concepts (6 questions), clinical information (5 questions), additional-complementary treatments (2 questions), nutrition management (15 questions), physical activities (4 questions), personal activities (4 questions), and male reproductive structure (4 questions). At the end of this questionnaire, an essay-type question was also included to provide the respondents with the opportunity to maintain their opinions and suggestions not covered by the questionnaire. The validity of the questionnaire was confirmed by a group of experts in medical informatics (n = 2), infertility (n = 2), and nutrition (n = 1). According to Cronbach’s test (alpha = 86.4), the questionnaire’s reliability was corroborated.

The target population of this research contained all men suffering from infertility and the research community included all nutrition and infertility specialists working at Yazd Shahid Sadoughi Infertility Center (one of the most equipped centers in Iran geographically located in the central area of the country that provides easier access for patients from different cities with various customs and cultures) and Rouyesh Red Crescent Clinic (another well-known and well-equipped center in the capital of Iran, Tehran).

Of the research community, 35 specialists (20 in infertility and 15 in nutrition) were purposefully selected. The criteria for entering the study were having at least seven years of experience working with infertile men and full cooperation with the above-mentioned centers. The exclusion criteria included lack of full cooperation, incomplete questionnaire, and lack of access to the participant due to lack of time, illness, and travel.

In the second phase, the designed questionnaire was administered and data were analyzed. Followed by designing and distributing the questionnaire among the experts, they were provided with clear explanations about the research purpose, confidentiality of information, as well as voluntary participation in the study to increase the rate of participation and compliance with the ethical standards. After obtaining the participants’ consent, questionnaires were distributed and collected after one week.

Data analysis was performed using descriptive statistics (mean and standard deviation) via SPSS version 24. The scores obtained from each item were added and the item was considered essential if it could receive 70% of the mean score (3.5 ≥ scores).

## Results

Of 35 distributed questionnaires, a total of 30 experts in the fields of infertility (n = 18) and nutrition (n = 12) completed and submitted the questionnaires. Table 1 illustrates the participants’ demographic information.

According to the collected data, 60% of the participants were nutritionists and 53% of them were employed in Rouyesh Red Crescent Clinic. Most participants were male (66%), were in the age range of 50 years and higher (46%), and had 10–12 years of work experience (43%).

The researcher-made questionnaire was designed in three main sections of the patient’s demographic information, educational content, and features of the designed application software (Tables 2, 3 and 4). According to Table 2, all items included.

in the questionnaire, except job experience and history of chronic diseases were confirmed and deemed necessary.

**Table 1:** Demographic information of the experts taking part in the study

Demographic/ information		Nutritionist (n = 18)				Specialist and Infertility specialist (n = 12)					Total	
		Sadoughi Yazd		Rouyesh Red Crescent		Sadoughi Yazd		Rouyesh Red Crescent				
		N	%	N	%	N	%		N	%	N	%
Age (years)	<40	2	6.7	1	3.3	1	3.3		0	0	4	13.3
	40–50	2	6.7	5	16.7	3	10		2	6.7	12	40
	>50	4	13.3	4	13.3	2	6.7		4	13.3	14	46.7
Gender	Female	3	10	4	13.3	1	3.3		2	6.7	10	33.3
	Male	5	16.7	6	20	5	16.7		4	13.3	20	66.7
Work experience (years)	7–9	1	3.3	2	6.7	1	3.3		1	3.3	5	16.7
	10–20	3	10	5	16.7	3	10		2	6.7	13	43.3
	>20	4	13.3	3	10	2	6.7		3	10	12	40

**Table 2:** Frequency distribution of participants’ responses regarding patients’ demographic information

Row	Demographic data / Responses	Mean/ Std. Deviation	Status
1	The patient’s age	3.76 ± 0.75	accept
2	Height	3.28 ± 0.8	accept
3	Weight	3.79 ± 0.72	accept
4	Economic status	4.32 ± 0.53	accept
5	Educational level	3.76 ± 0.45	accept
6	Field of study	3.46 ± 1.02	accept
7	Occupation	3.38 ± 0.6	reject
8	Residential region	3.9 ± 0.65	accept
9	History of chronic diseases	3.4 ± 0.9	reject

Table 3 is related to the educational items consisting of seven sections. All items related to clinical information sub-sections, additional-complementary treatments, physical activities, personal activities, and male reproductive structure were deemed necessary. However, only two items of ‘infertility effects’ from the main disease concepts section and ‘food allergy’ from the nutrition management section were not considered necessary by the participants.

Based on Table 4, all items related to the necessary features of the application software were approved and recognized as necessary by the experts.

**Table 3:** Frequency distribution of participants’ responses regarding educational items

Row	Educational content/Responses		Mean and standard deviation	Status
1	Main concepts of the disease	Defining infertility	4.4 ± 0.45	accept
		The effects of infertility	3.3 ± 1.02	reject
		Causative factors of infertility	3.8 ± 0.72	accept
		Effective factors on infertility (lifestyle, disease…)	4.05 ± 0.68	accept
		The effect of other diseases on infertility	3.7 ± 0.86	accept
		Types of treatment ways	3.9 ± 0.24	accept
2	Clinical information	Pharmaceutical treatment	4.1 ± 0.79	accept
		The effect of used medicines	3.8 ± 0.92	accept
		Treatment complications	3.7 ± 49	accept
		Genetic and innate influence	4.1 ± 0.41	accept
		Hormonal disorders	3.8 ± 0.53	accept
3	Additional treatments (complementary).	Herbal Medicines	4.2 ± 0.64	accept
		Alternative therapies (acupuncture)	4.1 ± 0.71	accept
4	Nutrition management	Nutritional habits	4.4 ± 0.48	accept
		Weight management	4.6 ± 0.26	accept
		Food diets (Mediterranean, Western, etc.	4.1 ± 0.70	accept
		Consuming carbohydrates	4.3 ± 81	accept
		Consuming Protein	4.1 ± 0.6	accept
		Consuming Fat	4.1 ± 0.43	accept
		Consuming Antioxidants	4.3 ± 76	accept
		Food allergy	3.4 ± 1.2	accept
		Consumption of micronutrients (vitamines, supplements (iron, zinc, vitamin D, etc.)	4.1 ± 0.69	accept
		Intaking fast food ready-to-eat foods	3.9 ± 0.84	accept
		Drinking tea	4.10 ± 0.64	accept
		Drinking coffee	4.18 ± 0.48	accept
		Drinking sweetened beverages	3.95 ± 0.70	accept
		The amount of sugar intake	4.05 ± 0.74	accept
		Drinking alcohol	3.57 ± 1.20	accept
5	Physical activities	Types of exercises (martial, movement, group, single)	4.08 ± 0.96	accept
		Exercising time	3.98 ± 0.84	accept
		Exercising Period	3.87 ± 0.61	accept
		Exercising method	4.05 ± 0.47	accept
6	Personal activities	Patient’s occupation	4.5 ± 0.38	accept
		Abusing drugs	4.36 ± 0.75	accept
		Personal hobbies and amusements	4.07 ± 0.83	accept
		Physical disabilities and problems	4.29 ± 0.57	accept
7	male reproductive structure	Number of sperms	4.17 ± 0.87	accept
		Quality of sperms	3.97 ± 0.36	accept
		Effective factors on sperms	4.07 ± 0.65	accept
		Related diseases (Varicocele and sperm disorders)	4.08 ± 0.47	accept

**Table 4:** The frequency of the participants’ answers on the application features

Row	Responses/ Qualities	Mean and standard deviation	Status
1	Reminding the appointment with the physician	3.88 ± 0.89	accept
2	Reminding the medication intake	4.14 ± 0.41	accept
3	Reminding performing the tests	3.7 ± 1.27	accept
4	Calculating BMI	3.97 ± 0.73	accept
5	The ability to write to a doctor	4.37 ± 0.46	accept
6	The ability to show movies and animations	4.06 ± 0.81	accept
7	Introducing infertility centers near the patient	3.57 ± 1.47	accept

In the final essay-type questions (asking for further suggestions), the participants mentioned some points summarized and categorized as the psychological dimensions, the social and religious dimensions, and the introduction of reliable infertility centers near the patient’s residential area.

## Discussion

Improvement of the patients’ educational knowledge and awareness will provide them with the opportunity to pay more attention to and develop a better understanding of their conditions to manage their disease and behavior more efficiently. In this study, based on the Literature review and guidelines, the items obtained after summarizing were prepared in the form of a researcher-made questionnaire, and after determining the validity and reliability, they were given to experts to confirm their necessity. The results showed that 39 cases were confirmed in the form of 7 categories (main disease concepts, clinical information, additional-complementary treatments, nutritional management, physical activities, personal activities and male reproductive structure). Also, all the features of the application program were recognized as necessary.

.

Hammarberg et al. [35]evaluated the desirable and necessary information regarding the factors affecting the chance of pregnancy in Australia and found that the participants neither perceived the factors affecting fertility nor paid much attention to the effect of age on fertility problems.

Harper et al. [36]also evaluated the effect of awareness on the chance of fertility and conception. To this end, a group of experts from different fields established an International Fertility Education Initiative (IFEI) entailing Asian countries and more than 20 European countries. Their findings clarified that the couples’ level of awareness about factors affecting infertility was very low and the need for education was vital. According to these scholars, the increase in age (especially in developed and high-income countries), the social and economic status (gender equality, lack of support policies, the family, increasing women’s education, etc.), and the chance of fertility through IVF were among the influential factors. In a cross-sectional descriptive study, Nagórska et al. [37] investigated health-related behaviors of infertile patients undergoing treatment in three clinics in southeastern Poland. They concluded that infertility decreased life satisfaction but deteriorated mental and emotional disorders among couples.

Ebrahimzadeh-Zagami et al [38]. conducted a review study with the aim of identifying the needs of infertile couples after unsuccessful treatment with assisted reproductive methods., which was in line with the present study, but the needs of support and counseling, psychology and social support, etc., were not mentioned in the present study.

In the United Kingdom, Stevenson et al. [22]studied the impact of male factors on infertility and noted that only half of the study population had sufficient information about the concept of fertility, which can be attributed to the complex clinical condition of infertility as a disease. They also found that women had more detailed and higher levels of information compared with men. These findings were in line with our results highlighting the need for infertility education, especially among men, according to the culture and geographical location of the patients’ residential area.

Aghdak, et al. [39] Conducted a study with the aim of determining the educational needs related to infertility. The results of this study were reported in the form of 21 priorities, which included the general sections of education about pregnancy, various diseases, prevention methods and various complications, and lifestyle, etc., which were considered in the present study.

Leisegang and Dutta [40] investigated the effect of lifestyle on male infertility and noted obesity and metabolic syndrome, alcohol and tobacco use, exposure to extreme heat, occupation and its location, inactivity, metabolic and endocrine disorders, drug abuse, exposure to radiation, and mental stress as effective factors on male’s infertility. These findings emphasized recording the patient’s history of the disease and lifestyle in the clinical treatment of infertility in order to determine and modify the patients’ inappropriate lifestyles.

Pedro et al. [41] conducted a systematic review to examine fertility awareness. They classified the effective factors on infertility into two categories of individual risk factors (such as obesity, lifestyle factors, drug abuse, and age) and non-individual factors (such as environmental factors and workplace conditions). They asserted the role of education and awareness, especially in the category of “personal related risk factors”.

All these studies reported almost similar effective factors on male infertility while the variety in the findings can be related to the number of participants, research design, research environment, the participants’ race, and geographical location of the study.

Among the limitations of this study was the lack of cooperation on the part of some experts due to their busy schedules or lack of personal interest. In this regard, the researcher tried to talk to these experts to explain the study’s aim and significance. Furthermore, a one-week deadline was considered for the respondents to submit their questionnaires. Due to the conditions of the coronavirus epidemic, only two infertility centers were considered, which is another limitation of this study.

## Conclusion

The rate of infertility, as a threat to the lives of couples, is increasing all over the world and Iran is no exception. In this vein, individuals with infertility need appropriate educational training, especially men. So, the present study was conducted with the aim of determining the required self-care training information for men with infertility in order to design a mobile phone-based application. The educational contents were categorized under seven areas, which can also be employed in designing other information systems such as decision support systems, and registry preparation. In addition, these educational contents are beneficial for the health care treatment staff and even the community members to increase their awareness.

### Electronic supplementary material

Below is the link to the electronic supplementary material.

Supplementary Material 1

## Data Availability

The data used and analyzed during the current study are not publicly available due Shahid Sadoughi University of Medical Sciences policy but are available from the corresponding author on reasonable request.
