# Supplementary material for: Determining the content and needs assessment a mobile-based self-care program in infertile men
Source: BMC Med Inform Decis Mak. 2023 Nov 13;23:258. doi: 10.1186/s12911-023-02366-2 (PMC10644630; doi:10.1186/s12911-023-02366-2)
Supplement: Supplementary file 1 — Supplementary Material 1 [file 12911_2023_2366_MOESM1_ESM.docx]

Dear respondents,

This questionnaire is designed to identify the necessary items for designing a conceptual model of self-care application program for infertile men. The questions are organized in different sections and have two Agree and Disagree answers.

Accurate response will help us to achieve research goals and correct results. The time to answer the questionnaire is about 25 to 30 minutes. If you have any questions, please contact Mr. Alireza Fallahzadeh. Thank you for your valuable time.

Alireza Fallahzadeh

Senior expert in Library and information science

Email: [afallahzadeh71@gmail.com](mailto:afallahzadeh71@gmail.com)

**A: Demographic Information**

Gender: Male Female

Age: <40 40-50 >50

Specialty: Nutritionist Infertility

Work Experience: 7-9 10-20 >20

Workplace: Rouyesh Red Crescent Sadoughi Yazd

B: Data Requirements items

B.1 Demographic Information

| Row | Information requirements | very low | low | moderate | high | very high |
| --- | --- | --- | --- | --- | --- | --- |
| 1 | The patient’s age |  |  |  |  |  |
| 2 | Height |  |  |  |  |  |
| 3 | Weight |  |  |  |  |  |
| 4 | Economic status |  |  |  |  |  |
| 5 | Educational level |  |  |  |  |  |
| 6 | Field of study |  |  |  |  |  |
| 7 | Occupation |  |  |  |  |  |
| 8 | Residential region |  |  |  |  |  |
| 9 | History of chronic diseases |  |  |  |  |  |

B.2: Educational information required

B.2.1: Main concepts of the disease

| Row | Information requirements | very low | low | moderate | high | very high |
| --- | --- | --- | --- | --- | --- | --- |
| 1 | Defining infertility |  |  |  |  |  |
| 2 | The effects of infertility |  |  |  |  |  |
| 3 | Causative factors of infertility |  |  |  |  |  |
| 4 | Effective factors on infertility (lifestyle, disease...) |  |  |  |  |  |
| 5 | The effect of other diseases on infertility |  |  |  |  |  |
| 6 | Types of treatment ways |  |  |  |  |  |

B.2.2: clinical information

| Row | Information requirements | very low | low | moderate | high | very high |
| --- | --- | --- | --- | --- | --- | --- |
| 7 | Pharmaceutical treatment |  |  |  |  |  |
| 8 | The effect of used medicines |  |  |  |  |  |
| 9 | Treatment complications |  |  |  |  |  |
| 10 | Genetic and innate influence |  |  |  |  |  |
| 11 | Hormonal disorders |  |  |  |  |  |

B.2.3: Additional treatments (complementary).

| Row | Information requirements | very low | low | moderate | high | very high |
| --- | --- | --- | --- | --- | --- | --- |
| 12 | Herbal Medicines |  |  |  |  |  |
| 13 | Alternative therapies (acupuncture) |  |  |  |  |  |

B.2.4: Nutrition management

| Row | Information requirements | very low | low | moderate | high | very high |
| --- | --- | --- | --- | --- | --- | --- |
| 14 | Nutritional habits |  |  |  |  |  |
| 15 | Weight management |  |  |  |  |  |
| 16 | Food diets (Mediterranean, Western, etc. |  |  |  |  |  |
| 17 | Consuming carbohydrates |  |  |  |  |  |
| 18 | Consuming Protein |  |  |  |  |  |
| 19 | Consuming Fat |  |  |  |  |  |
| 20 | Consuming Antioxidants |  |  |  |  |  |
| 21 | Food allergy |  |  |  |  |  |
| 22 | Consumption of micronutrients (vitamines, supplements (iron, zinc, vitamin D, etc.) |  |  |  |  |  |
| 23 | Intaking fast food ready-to-eat foods |  |  |  |  |  |
| 24 | Drinking tea |  |  |  |  |  |
| 25 | Drinking coffee |  |  |  |  |  |
| 26 | Drinking sweetened beverages |  |  |  |  |  |
| 27 | The amount of sugar intake |  |  |  |  |  |
| 28 | Drinking alcohol |  |  |  |  |  |

B.2.5: physical activities

| Row | Information requirements | very low | low | moderate | high | very high |
| --- | --- | --- | --- | --- | --- | --- |
| 29 | Types of exercises (martial, movement, group, single) |  |  |  |  |  |
| 30 | Exercising time |  |  |  |  |  |
| 31 | Exercising Period |  |  |  |  |  |
| 32 | Exercising method |  |  |  |  |  |

B.2.6: personal activities

| Row | Information requirements | very low | low | moderate | high | very high |
| --- | --- | --- | --- | --- | --- | --- |
| 33 | Patient's occupation |  |  |  |  |  |
| 34 | Abusing drugs |  |  |  |  |  |
| 35 | Personal hobbies and amusements |  |  |  |  |  |
| 36 | Physical disabilities and problems |  |  |  |  |  |

B.2.7: male reproductive structure

| Row | Information requirements | very low | low | moderate | high | very high |
| --- | --- | --- | --- | --- | --- | --- |
| 37 | Number of sperms |  |  |  |  |  |
| 38 | Quality of sperms |  |  |  |  |  |
| 39 | Effective factors on sperms |  |  |  |  |  |
| 40 | Related diseases (Varicocele and sperm disorders) |  |  |  |  |  |

C: Technical Capabilities

| Row | Information requirements | very low | low | moderate | high | very high |
| --- | --- | --- | --- | --- | --- | --- |
| 1 | Reminding the appointment with the physician |  |  |  |  |  |
| 2 | Reminding the medication intake |  |  |  |  |  |
| 3 | Reminding performing the tests |  |  |  |  |  |
| 4 | Calculating BMI |  |  |  |  |  |
| 5 | The ability to write to a doctor |  |  |  |  |  |
| 6 | The ability to show movies and animations |  |  |  |  |  |
| 7 | Introducing infertility centers near the patient |  |  |  |  |  |

D:Please enter your suggestions below

………………………………………………………………………………………………………………………………………………………………………………………………………………………………………………………………………………………………………………………………………………
